# Research Status and Trends in Periodontal Ligament Stem Cells: A Bibliometric Analysis over the Past Two Decades

**DOI:** 10.1155/2024/9955136

**Published:** 2024-09-27

**Authors:** Zhengyang Li, Jinyi Li, Shanshan Dai, Ruirui Liu, Qingyu Guo, Fei Liu

**Affiliations:** ^1^ Key Laboratory of Shaanxi Province for Craniofacial Precision Medicine Research College of Stomatology Xi'an Jiaotong University, Xi'an 710004, China; ^2^ Department of Pediatric Dentistry College of Stomatology Xi'an Jiaotong University, Xi'an 710004, China; ^3^ Department of Prosthodontics College of Stomatology Xi'an Jiaotong University, Xi'an 710004, China

## Abstract

**Objective:**

Currently, the summaries of research on periodontal ligament stem cells (PDLSCs) are mainly reviews, and the systematic evaluation of all relevant studies is lacking. The aim of our study was to reveal the research status and developmental trends of PDLSCs using bibliometric analyses.

**Methods:**

Publications on PDLSC from 2004 to 2023 in the PubMed database were searched and then screened according to certain inclusion and exclusion criteria. Two researchers browsed the included papers and recorded information such as the research type and research model. The VOSviewer software was used to analyze the distribution of authors, journals, and institutions. The contents and directions of PDLSC research were summarized by analyzing high-frequency keywords. The CiteSpace software was used to monitor burst words, determine hot factors, and indicate developmental trends.

**Results:**

During the past two decades, the number of studies on PDLSCs increased. China published the most related papers. The primary type of article was basic research. Among core journals, the *Journal of Periodontal Research* had the highest number of publications. The Fourth Military Medical University (China) was leading in the number of articles on PDLSCs. Research topics mainly included mechanism of periodontal diseases, tissue engineering and regeneration, biological characteristics of PDLSCs, and comparison with other stem cells. Infectious inflammation and mechanical stimulation were important pathological conditions and research topics.

**Conclusion:**

The research of PDLSCs is still in a rapid development stage. Our study provides new insights into the current research status and future trend in this field.

## 1. Introduction

Periodontal ligament (PDL) contains a type of adult stem cell with proliferation and differentiation potential in vitro, which plays an important role in maintaining the stability of the periodontal system through tissue repair and regeneration. Previous studies have shown that periodontal ligament stem cells (PDLSCs) have unique advantages in periodontal tissue engineering, as they can express markers of osteoblasts/cementoblasts during in vitro induction [1] and form typical cementum/PDL-like structures during in vivo transplantation [2]. The accessibility, plasticity, and high proliferative capacity of PDLSCs have attracted extensive attention from researchers. They play an important role not only in the repair of oral tissues but also in many other fields of tissue engineering and regenerative medicine [3]. In addition, as fewer ethical disputes are associated with the studies and applications of PDLSCs, they are expected to become an important direction in regenerative medicine research.

Since the isolation and identification of PDLSCs firstly in 2004, rapid progress has been made in this field. Many achievements related to PDLSCs have emerged, raising the requirement for a comprehensive and systematic evaluation and summary of the current research progress. The source and characteristics of PDLSCs are important research aspects in the initial stage. Some researchers [4, 5] have discussed the clustering and identification of stem cells in PDL tissue based on previous experiments. The morphological and functional features, surface markers, and biological behaviors of PDLSCs, including self-renewal, multidirectional differentiation, and immunomodulatory effects [6, 7], have been summarized and reported in many articles. In addition, the differences in biological characteristics between PDLSCs and mesenchymal stem cells (MSCs) derived from other tissue were compared by some scholars [8, 9].

Studies on the pathogenesis and treatment of diseases related to PDLSCs are also an important direction in this field. Zhang et al. [10] elucidated the interactions of PDLSCs with the periodontal microenvironment and provided novel interventions for periodontitis. Huang et al. [11] discussed the PDLSC fate to physical force in vitro and orthodontic force in vivo, as well as the underlying molecular mechanism involved in orthodontic tooth movement, which might provide ways for regulating biological effects induced by mechanical stress.

Applications of PDLSCs in tissue repair and regeneration have always appealed to researchers. Many reviews focus on the theoretical and technical challenges related to periodontal regeneration, including the therapeutic potential of PDLSCs [12], strategies for large-scale expansion in vitro [13, 14], and their combination with various bioactive materials, scaffolds, and growth factors [15, 16]. PDLSCs have made contributions even to the repair of other tissues [17]. Wang et al. [18] and Mohebichamkhorami et al. [19] reported on the neuronal differentiation potential of PDLSCs and their application value in neural regeneration. In addition, derivatives of PDLSCs have also become research hotspots recently. The composition, biological effects, and application potential of conditioned medium [20], exosomes [21], and extracellular vesicles [22] derived from PDLSCs have been summarized and reported in some articles.

Currently, the summaries of research on PDLSCs are mainly reviews, which present the results of representative literature and mostly focus on one specific aspect [7, 23], lacking the systematic evaluation of all relevant studies. Bibliometrics, as a scientific and objective method of literature investigation, explores the relationship between articles and research entities through the employment of quantitative analysis methods, such as mathematics and statistics, reflects the development of specific disciplines, and has been widely used in various fields of medical research. Bibliometric analyses of stem cells involve a variety of cell sources, including umbilical cord mesenchymal stem cells [24], adipose stem cells [25], and hair follicle stem cells [26]. Zhao et al. [27] revealed the current research status and trends in dental stem cells by conducting quantitative analysis on the number of publications, time, authors, journals, institutions, regions, research topics, and keywords. Yan and Su [28] carried out a statistical analysis on research related to dental pulp stem cells. At present, bibliometric analyses on periodontal ligament stem cells are still lacking, and the scientific evaluation of the progress in this field is relatively insufficient.

This study aimed to search for relevant studies on PDLSCs in the past two decades and summarize and analyze the distribution characteristics and structural relationships of these publications using bibliometric methods to understand the research status and hot frontiers of PDLSCs and inspire future studies.

## 2. Materials and Methods

### 2.1. Search Strategy

Publications in research of PDLSCs were searched for in PubMed database, and the search formula was set as follows: (periodontal ligament stromal cell) OR (periodontal ligament stem cell) OR (periodontal ligament-derived mesenchymal stromal cell) OR (periodontal ligament-derived mesenchymal stem cell). The time period of publications was from 2004.01.01 to 2023.12.31. Relevant articles were selected according to the following criteria to determine the research objectives: Inclusion criteria included clinical and basic research based on PDLSCs and review literature related to PDLSCs. Exclusion criteria included research unrelated to the research topic, non-English language, letters, comments, and articles without complete information, such as inaccessible abstract or full text.

### 2.2. Data Collection

Two researchers preliminarily screened the retrieved articles by evaluating the title and abstract and browsing the full text if necessary. Concomitantly, they recorded the type of articles (basic research, reviews, case reports, clinical trials, etc.) and established cell models and organized the obtained information. If disagreement was present, a third investigator assisted in resolving the discrepancy after discussion. The included articles were searched again on the Web of Science, and their complete records were exported as plain text for visualizing bibliometric networks.

### 2.3. Statistical Analysis

The time trend of the number of publications was analyzed using a mathematical fitting curve via GraphPad Prism 8. Data visualization and analysis software, such as VOSviewer and CiteSpace, were further used to conduct various analyses, such as co-authorship network analysis, co-occurrence analysis, and mutated word analysis of the included literature. Synonyms were merged during the keyword analysis.

## 3. Results

### 3.1. Search and Screening of Literature

**Figure 1:**
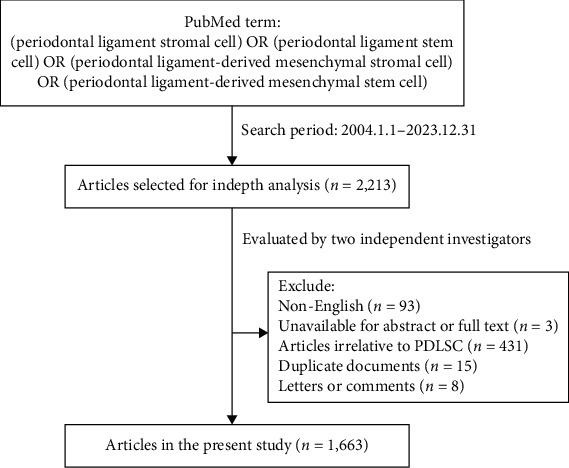
Flow diagram of the process of selection of articles.

Using our established strategy, 2,213 articles were initially retrieved. Further screening was conducted based on the inclusion and exclusion criteria and 1,663 articles related to PDLSCs were ultimately included (Figure 1).

### 3.2. Spatiotemporal Distribution of Literature

**Figure 2:**
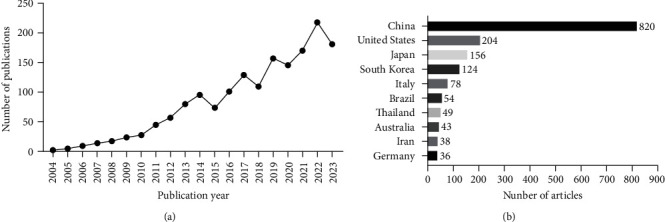
Distribution characteristics of literature according to publication years and countries: (a) annual distribution trend of articles on PDLSCs and (b) top 10 countries with the highest number of publications on PDLSCs.

During the past two decades, the number of articles related to PDLSCs increased in a fluctuating way (Figure 2(a)). In particular, the average annual number of publications from 2004 to 2013 was 28, which increased nearly fivefold from 2014 to 2023 (138.3). Among the countries and regions contributed to research on PDLSCs, China published the largest number of related papers (820 articles, 49.31%), followed by the United States (204 articles, 12.27%) and Japan (156 articles, 9.38%) (Figure 2(b)).

### 3.3. Journal Distribution of Literature

The 1,663 articles included in the study were published in 422 journals. All these journals were arranged in descending order based on the number of relevant papers published. The top 15 journals accounted for 32.89%, approximately one-third, of the total number of articles (547/1663) (Table 1), which met the standards of core journals. Nearly half of these journals were published in the United States. More specifically, the most articles were published in the *Journal of Periodontal Research*, and the relevant publications in *Journal of Dental Research* had the highest number of citations.

### 3.4. Distribution of Authors

**Figure 3:**
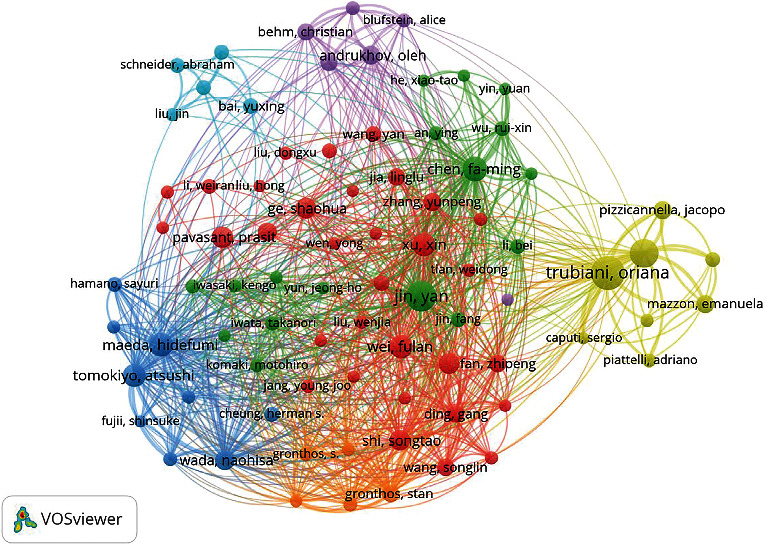
Co-authorship network map of authors working on PDLSCs. The size of the nodes represents the number of publications, while the thickness of the link represents the cooperation strength. The colors represent different clusters which are determined by link strength between authors.

Among the top 10 authors in terms of the number of publications (Table 2), Trubiani and Oriana from Italy published the most studies on PDLSCs, with a total of 46 articles (2.77%), followed by Jin Yan from China (39 articles, 2.35%) and Diomede and Francesca from Italy (36 articles, 2.16%). According to the Price Law, 82 researchers constituted the core group of authors in the research field of PDLSCs in this analysis.  Figure 3 shows the number of publications from each author and the cooperative relationships among them. The results indicated that 82 core authors formed a complex co-authorship network, with authors from the same region having stronger link strength.

### 3.5. Institution Distribution of Literature

**Figure 4:**
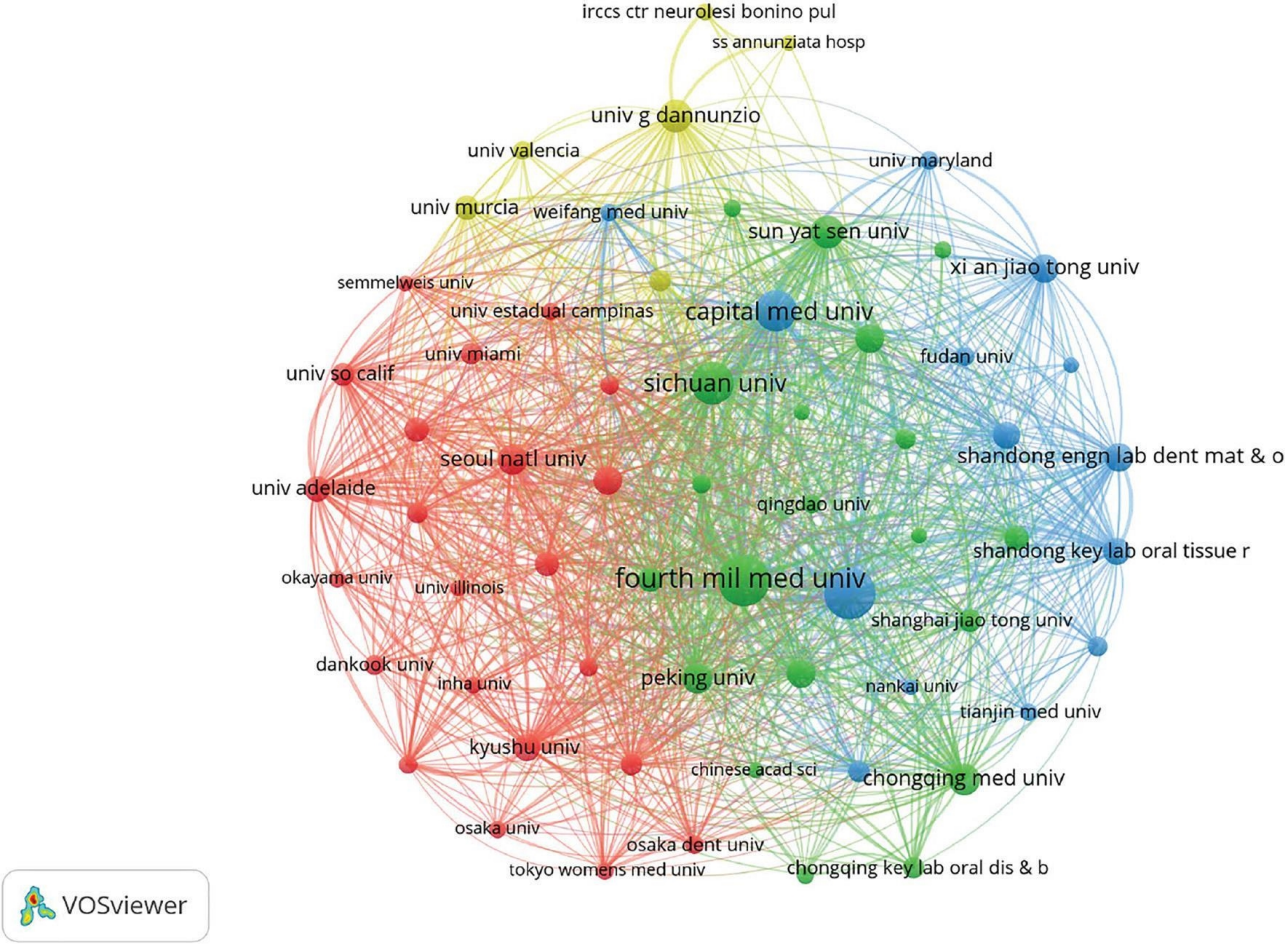
Mapping institutional co-authorship network of publications on PDLSCs. The size of each node represents the number of publications from each institution. The thickness of the line indicates the collaboration degree between two institutions.

As shown in Table 3, the institutions with the greatest contribution on research of PDLSCs was the Fourth Military Medical University (China), followed by Shandong University (China), both of which produced more than 100 articles. Moreover, among the top 10 institutions, Seoul National University had the highest total and average citation frequencies (4,994 and 131 citations, respectively). A co-authorship analysis was conducted on institutions that published more than 10 articles, respectively. These institutions formed the largest institutional co-authorship network and were divided into four clusters (Figure 4). The cluster with the largest number of publications (a total of 581 articles, 34.94%) consisted of 20 institutions, mainly located in China, with the Fourth Military Medical University, Sichuan University, and Sun Yat Sen University as the core. Twenty-two institutions, including Seoul National University in South Korea, Chulalongkorn University in Thailand, and Kyushu University in Japan, constituted the second largest cluster, with 413 published articles (24.83%). Notably, these institutions cooperated closely with each other and made outstanding contributions to the research on PDLSCs.

### 3.6. Word Frequency Analysis

**Figure 5:**
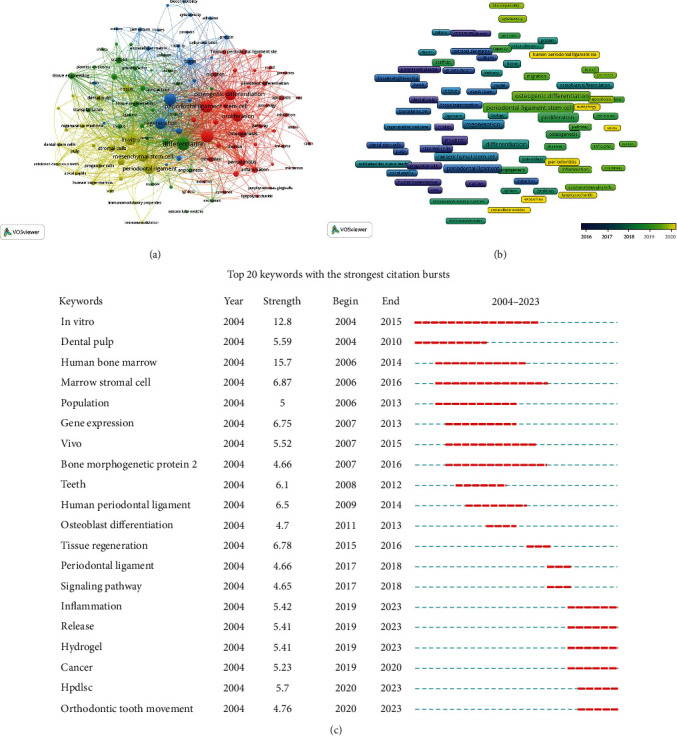
Analysis of keywords in publications related to periodontal ligament stem cells. (a) Co-occurrence network map of high-frequency keywords. Keywords are grouped into four clusters indicated by different colors. Large nodes represent keywords with high frequencies. (b) Distribution of high-frequency keywords according to the average publication year. The color of the nodes represents the time of appearance. (c) Top 20 keywords with the strongest citation bursts. The red segment of the blue line denotes the burst duration of a keyword.

Co-occurrence and cluster analyses were conducted on the high-frequency keywords from the included documents using the VOSviewer software (Figure 5(a)). According to correlation strength, the keywords could be divided into four clusters, and the representative ones of each cluster were listed in Table 4. Then the main research topics were summarized as follows: mechanism of periodontal diseases (red cluster), tissue engineering and regeneration (green cluster), biological characteristics of PDLSCs (blue cluster), and comparison with other stem cells (yellow cluster).

We also conducted a visualization analysis to determine the average year in which high-frequency keywords appeared. The color of the nodes represented the time of appearance, with blue color-coded keywords indicating keywords from early publications and yellow nodes representing the most recent keywords. As shown in Figure 5(b), the research contents in this field had gradually shifted from “sources and characteristics of stem cells” to “disease mechanisms and applications related to PDLSCs.”

The CiteSpace software was further used to analyze the variation patterns of mutated words in the included literature. The top 20 keywords with the strongest citation bursts shown in Figure 5(c) were arranged in chronological order, and the trend of changes in the research topics presented by them was consistent with the results shown in Figure 5(b). The most recent mutated words included “inflammation,” “cancer,” “orthodontic tooth movement,” “signaling pathway,” “tissue regeneration,” “hydrogel,” and “release.” It could be concluded that “the mechanisms of related diseases” and “application of tissue regeneration” were the main research hotspots in the field of PDLSCs during the past decade.

### 3.7. Types of Literature

As shown in Table 5, the vast majority of the 1,663 articles were basic studies (1,410 articles, 84.79%), followed by review studies (237 articles, 14.25%); the number of clinical trials and case reports was relatively small. A subanalysis on the types of review studies was further conducted to understand the distribution status of bibliometrics. Among the review articles, the most common types were reviews and systematic reviews, accounting for 13.71% of the total studies. Meta-analyses (eight articles, 0.48%) and bibliometrics (one article, 0.06%), which were either quantitative or semiquantitative studies, accounted for a relatively small proportion.

### 3.8. Research Model of Cells in Literature

Based on the research model of PDLSCs established in the experiments, basic studies could be divided into two categories: cells in physiological state or in pathological state. Among them, 989 articles explored the biological characteristics of PDLSCs or intervention effects of drugs and compounds on PDLSCs under physiological conditions, while 421 articles mainly reported research on PDLSCs under pathological conditions by establishing disease models or extracting cells from pathological tissues.

According to the different pathology types studied, articles reporting on the pathological status of PDLSCs were divided into the following categories: infectious inflammation, mechanical stimulation, hypoxia, oxidative stress, and systemic diseases, including diabetes, smoking, bisphosphonate-related osteonecrosis of the jaw, and multiple sclerosis. Among them, infectious inflammation, represented by periodontitis, accounted for the highest proportion (231 articles, 54.87%), followed by mechanical stimulation (81 articles, 19.24%), indicating that these pathological types and research directions received more attention (Table 6).

## 4. Discussion

Bibliometric analysis is a scientific way of literature investigation that uses statistics, mathematics, and other methods to present the quantitative relationships and distribution of published articles. It is an effective tool for revealing the research status and development trends of specific disciplines. To understand the research status of PDLSCs more comprehensively, the PubMed database that focuses on biomedical fields was selected for document retrieval in this study. Concomitantly, the Web of Science was used to obtain citation information to scientifically evaluate the structural relationships and distribution characteristics of the literature.

The annual trend in the number of publications is an effective indicator of the development of a specific discipline [29]. With this search formula, it could be found that the number of published articles before 2023 was 2,273, whereas from 2004 to 2023, the number was 2,213, accounting for 97.36% of the total. Besides, PDLSCs were firstly isolated and identified in 2004. Therefore, relevant articles published between 2004 and 2023 were representative in this field and thus selected as the research objects. Our results indicated that the number of publications related to PDLSCs increased in a fluctuating way during the past 20 years. The slope of the year publication curve shows an increasing trend, indicating the increased attention given to the research on PDLSCs.

The geographical distribution of published studies reflects the differences in scientific and technological development and research capabilities among countries to a certain extent [30]. In this study, China had the highest number of publications and citation frequency, making significant contributions to research in this field, which might be related to its policy orientation, emphasis, and investment in scientific research.

Bibliometrics can also be used for the evaluation of journals and institutions. In this study, seven of the 15 core journals are published in the United States, accounting for nearly half the total, followed by the United Kingdom. Being technological powers, both the United States and United Kingdom occupied important positions in mainstream international journals. The citation frequency and impact factor, two major measurement indicators for journal evaluation, reflect journal quality and academic influence [31]. The *Journal of Dental Research* was the most cited and had the highest impact factor during the past 5 years, indicating its significance and reference value in the research of PDLSCs. Analysis of institutions revealed that the Fourth Military Medical University and Shandong University from China were in the leading positions in the number of publications, being the most productive institutions in the research field of PDLSCs from 2004 to 2023. Importantly, universities and colleges have emerged as the main forces of scientific research among numerous institutions.

The distribution of authors is closely related to the development of academic fields. Statistical analysis of the author groups conducting PDLSC research can predict and reveal the abilities of researchers in this field. Our results indicated that the authors studying PDLSCs were relatively concentrated, forming an influential group of core authors. Some scholars have conducted more in-depth and sustained studies. According to our co-authorship network analysis, the top 10 productive authors were located in the areas with the densest correlation and nodes distribution, reflecting their important role in PDLSC research. Most of these authors came from affiliated university hospitals with access to abundant academic resources and advanced experimental conditions. Support from dependent units and disciplines, superior platforms, and talent environments are crucial for cultivating leading researchers [32].

Coword analysis is used to identify research hotspots and development trends in a certain discipline, guiding scholars in conducting further exploration. The research topics on PDLSCs were divided into the following four categories according to keyword clustering.

1. Mechanisms of periodontal diseases. Periodontal disease has a high incidence rate and prevalence, and the World Health Organization has listed periodontal health as one of the 10 standards of human health [33]. The mechanism of periodontal disease has attracted widespread attention from scholars worldwide. The main keywords in this cluster were “expression,” “osteogenesis,” “proliferation,” “periodontitis,” “inflammation,” and “pathway,” indicating that inflammation was the main type of disease in the periodontal system. The biological characteristics of PDLCs, such as cell proliferation and differentiation, and changes in the expression levels of gene and protein, were the main directions of mechanistic research. The distribution of keywords according to the average publication year showed that “exosomes,” “extracellular vesicles,” “stress,” and “autophagy” appeared relatively recently and became research hotspots in the study of the mechanism of periodontal disease in the recent years.
2. Tissue engineering and regeneration. The regenerative properties of PDLSCs make them an excellent source of cells for tissue engineering applications. In addition to “differentiation” and “tissue regeneration,” the representative keywords in this cluster also included “scaffold,” “growth factors,” “transplantation,” “release,” and “biomaterials,” indicating that the improvement of scaffold materials and combined application of growth factors were the main research directions in tissue engineering and regeneration. Currently, in addition to their excellent mechanical and biological properties, scaffold materials are also combined with various pharmaceuticals for the loading and release of antibiotics [34], metformin [35], aspirin [36], and other drugs with anti-inflammatory and bone-promoting effects to improve the regeneration of periodontal tissue in the inflammatory environment. Moreover, the application of growth factors has gradually diversified, shifting from single components (FGF, TGF-*β*, etc.) to natural composite components, such as extracellular vesicles [37], enamel matrix derivatives [38], and platelet lysates [39], which help provide a growth-favorable environment for cells.
3. Biological characteristics of PDLSCs. Migration, proliferation, differentiation, and other biological behaviors are important aspects of cell state, and the corresponding regulatory mechanisms are the main focus of research on the characteristics of PDLSCs. With the development of material technology, new biomaterials are constantly emerging, and PDLSCs are often used to evaluate the biological safety of such materials. Keywords such as “cytotoxicity,” “adhesion,” and “biocompatibility” were relatively popular in the included studies, indicating that dental materials may become an important direction in future research, with studies on PDLSCs gradually transitioning toward the field of translational applications.
4. Comparison with other stem cells. Most researchers select bone marrow mesenchymal stem cells as the main source of adult stem cells for tissue regeneration research. However, as the exploration of dental stem cells deepens, the use of stem cells obtained from the periodontal ligament, dental pulp, dental papilla, and dental sac has gradually been explored in various studies. Several comparative experiments have shown that stem cells from different tissue sources exhibit heterogeneity in cytokine expression, immune regulatory effects, and cell proliferation and differentiation ability [40, 41, 42]. Dental stem cells are more adaptable to inflammatory environments than MSCs derived from other tissues. Transplantation of PDLSCs can promote the regeneration of periodontal ligament and cementum, forming a more typical structure of the cementum/periodontal ligament/alveolar bone, thus indicating PDLSCs as the most suitable seed cells for periodontal regeneration therapy [43, 44]. As MSCs inherit the characteristics of their originating tissue [45], standardized research on in vitro culture systems and stem cell sources can guide more accurate clinical applications of MSCs in the future.

The mutated words focus on active factors with potential influence in a certain research field, which can reflect changes in local research hotspots and effectively indicate the development direction of a specific discipline. In this study, CiteSpace was used to monitor important mutated words in PDLSC research annually. The results showed that the most active hot factors in the past two decades included “sources and biological characteristics of stem cells,” “tissue regeneration,” and “mechanisms of related diseases,” which were consistent with the main directions of PDLSC research concluded from the co-occurrence analysis of high-frequency keywords.

Studies on PDLSCs were mostly basic studies that explored the cell characteristics, regulation mechanisms, and interventional effects under physiological or pathological states through in vitro experiments or animal models. The safety and ethical issues associated with stem cell therapy have limited clinical research on PDLSCs [46]. A few studies have transplanted autologous PDLSCs into the bone defect area to observe improvement in clinical symptoms in patients with periodontal disease [47, 48]. However, the maintenance of stem cell characteristics, transplantation methods, timing, and indications require further clarification. In the future, it will be necessary to conduct more comprehensive and in-depth basic research on PDLSCs to achieve safe and effective clinical applications [7]. Among the review articles, only one bibliometric study was included that analyzed the research status and development trend of microRNAs in the field of periodontal disease and oral implantology [49] and paid more attention to the specific mechanism—the role of gene regulation in the pathogenesis and treatment of periodontal disease. An overall review and scientific evaluation of the relevant literature were conducted in our study to comprehensively understand the research status of PDLSCs in various fields.

According to the analysis of research models, two major pathological types, infectious inflammation and mechanical stimulation, have received increasing attention and been the focus of research on periodontal diseases. Periodontitis, which has a high incidence and prevalence, is the leading cause of tooth loss in adults. Periodontal ligament, the primary tissue responsible for bearing and buffering the mechanical stimuli of the periodontal system, is often exposed to various mechanical environments. Therefore, exploring the effects and mechanisms of inflammation and mechanical forces on PDLSCs is of great significance.

This study systematically summarized and analyzed academic articles in the research field of PDLSCs using bibliometric analyses; however, there were some limitations that could not be ignored. First, only a single database was searched, which did not cover all biomedical journals. Second, the exclusion of non-English articles may have overlooked important studies in other languages. Third, some recently published high-quality papers may have been neglected because of their short publication time and low citation frequency. Attention should be paid to the latest studies and non-English literature in the future, which will help to better and more comprehensively evaluate the current research status of PDLSCs.

## 5. Conclusions

During the past two decades, the number of studies on PDLSCs increased. The primary type of article was basic research. Research topics mainly included mechanism of periodontal disease, tissue engineering and regeneration, biological characteristics of PDLSCs, and comparison with other stem cells. Infectious inflammation and mechanical stimulation were important pathological conditions and research topics. Our study provides a fresh perspective of global PDLSCs research, enabling us to understand the past, present, and future.

## Figures and Tables

**Table 1:** Core journals of research on PDLSCs.

Rank	Journal	Publications (pieces/%)	Citations	Country	5-Y IF
1	*Journal of Periodontal Research*	68 (4.09)	1,813	Denmark	4.072
2	*Journal of Periodontology*	55 (3.31)	1,199	USA	5.277
3	*Archives of Oral Biology*	52 (3.13)	929	UK	2.687
4	*Stem Cells International*	46 (2.77)	792	USA	5.632
5	*Stem Cell Research & Therapy*	40 (2.41)	1,090	UK	8.387
6	*Oral Diseases*	39 (2.35)	704	Denmark	3.598
7	*Stem Cells and Development*	38 (2.29)	1,514	USA	4.114
8	*Scientific Reports*	36 (2.16)	811	UK	5.516
9	*International Journal of Molecular Sciences*	34 (2.04)	511	Switzerland	6.628
10	*Journal of Dental Research*	33 (1.98)	2,459	USA	8.463
11	*Tissue Engineering Part A*	26 (1.56)	1,053	USA	4.541
12	*Journal of Cellular Physiology*	24 (1.44)	1,181	USA	6.398
13	*Clinical Oral Investigations*	19 (1.14)	329	Germany	3.545
14	*PLOS One*	19 (1.14)	1,360	USA	4.069
15	*Cell Proliferation*	18 (1.08)	312	China	7.795

*Notes*. “5-Y IF” refers to “5-year impact factor.”

**Table 2:** Top 10 productive authors in the research field of PDLSCs.

Rank	Author	Articles (pieces/%)	Affiliation
1	Trubiani, Oriana	46 (2.77)	Department of Medical, Oral and Biotechnological Sciences, University “G. d'Annunzio” Chieti-Pescara, Chieti, Italy

2	Jin, Yan	39 (2.35)	Research and Development Center for Tissue Engineering, Fourth Military Medical University, Xi'an, China

3	Diomede, Francesca	36 (2.16)	Department of Medical, Oral and Biotechnological Sciences, University “G. d'Annunzio” Chieti-Pescara, Chieti, Italy

4	Chen, Fa-ming	28 (1.68)	Department of Periodontology and Oral Medicine, School of Stomatology, Fourth Military Medical University, Xi'an, China

5	Maeda, Hidefumi	27 (1.62)	Division of Endodontics, Kyushu University Hospital, Fukuoka, Japan

6	Tomokiyo, Atsushi	25 (1.50)	Department of Restorative Dentistry, Faculty of Dental Medicine, Hokkaido University, Sapporo 0608586, Japan

7	Wei, Fulan	25 (1.50)	Shandong Provincial Key Laboratory of Oral Tissue Regeneration, School of Stomatology, Shandong University, Jinan, China

8	Xu, Xin	25 (1.50)	Regeneration, School of Stomatology, Shandong University, Jinan, China

9	Pavasant, Prasit	23 (1.38)	Center of Excellence in Regenerative Dentistry, Faculty of Dentistry, Chulalongkorn University, Bangkok, Thailand

10	Ge, Shaohua	22 (1.32)	Shandong Provincial Key Laboratory of Oral Tissue Shandong Provincial Key Laboratory of Oral Tissue Regeneration, School of Stomatology, Shandong University, Jinan, China

10	Shi, Songtao	22 (1.32)	South China Center of Craniofacial Stem Cell Research, Guanghua School of Stomatology, Sun Yat-sen University, Guangzhou, China

**Table 3:** Top 10 contributive organizations with publications on PDLSCs.

Rank	Institutions	Counts	Citations	Average citations
1	Fourth Military Medical University	109	4,138	38
2	Shandong University	107	1,883	18
3	Sichuan University	77	1,369	16
4	Capital Medical University	69	2,839	41
5	Sun Yat Sen University	44	836	19
5	University “G. d'Annunzio”	44	1,523	35
7	Chongqing Medical University	41	470	11
8	Seoul National University	38	4,994	131
9	Peking University	37	1,716	46
10	Nanjing Medical University	35	699	20
10	Shandong Engineering Laboratory for Dental Materials and Oral Tissue Regeneration	35	315	9
10	Xi'an Jiaotong University	35	514	15
10	Zhejiang University	35	288	8

**Table 4:** Clustering results of keywords in publications related to PDLSCs.

Cluster	Research topic	Keywords
Red cluster	Mechanism of periodontal-related diseases	Inflammation, periodontitis, disease, expression, pathway, mechanisms, oxidative stress, osteogenesis, apoptosis, activation, inhibition, runx2, TNF-*α*

Green cluster	Tissue engineering and regeneration	Tissue engineering, tissue regeneration, differentiation, transplantation, repair, periodontal regeneration, bone regeneration, scaffold, biomaterials, growth factor

Blue cluster	Biological characteristics of PDLSCs	Stem cell, cell differentiation, cell proliferation, capacity, adhesion, fibroblasts, protein, extracellular matrix, identification, culture, cytotoxicity

Yellow cluster	Comparison with other stem cells	Mesenchymal stem cells, dental stem cells, periodontal ligament, tissue, dental pulp, exfoliated deciduous teeth, apical papilla, bone marrow, in vitro

**Table 5:** Types of articles on PDLSCs.

Research types	Publications (pieces/%)
Basic studies	1,410 (84.79)
Review studies	237 (14.25)
Case reports	4 (0.24)
Clinical trials	5 (0.30)
Others	7 (0.42)
Total	1,663 (100)

**Table 6:** Pathological types of cell models established in the studies of PDLSCs.

Pathological types	Publications (pieces/%)
Infectious inflammation	231 (54.87)
Mechanical stimulation	81 (19.24)
Hypoxia	18 (4.28)
Oxidative stress	14 (3.33)
Systemic diseases	59 (14.01)
More than one types	18 (4.28)
Total	421 (100)

## Data Availability

The datasets used and analyzed during the current study are available from the corresponding author on reasonable request.

## Abbreviations

PDLSCs: Periodontal ligament stem cells
PDL: Periodontal ligament
MSCs: Mesenchymal stem cells.

## Additional Points

The authors declare that they have not used generative AI and AI-assisted technologies in the writing process.
